# Bellis-Perennis

**Published:** 1895-11

**Authors:** Wm. G. Dietz

**Affiliations:** Hazleton, Pa.


### BELLIS-PERENNIS.

Editor Homœopathic Physician:

On page 473, October number of your journal, appears an article by Edmund Carleton, M. D., entitled "Accidental Provings of Bellis-Perennis." A perusal of this article leaves scarcely a doubt, that the plant in question, the common daisy, is not Bellis-perennis L., the common daisy of Europe, but Chrysanthemum leucanthemum L., our common daisy. Bellis-perennis is a low, herbaceous plant which, to the best of my knowledge, has not as yet found a permanent, if an occasional, resting-place on our shores. Both species are members of the same family, Compositæ, and are nearly related. While their pathogenetic effects, whatever they are, may be identical, strict observance of scientific nomenclature is a *sine qua non* in all reports of provings, whether by accident or intent.

Hazleton, Pa., Oct. 25th, 1895. Wm. G. Dietz, M. D.

